# Headache service quality: the role of specialized headache centres within structured headache services, and suggested standards and criteria as centres of excellence

**DOI:** 10.1186/s10194-019-0970-7

**Published:** 2019-03-04

**Authors:** T. J. Steiner, H. Göbel, R. Jensen, C. Lampl, K. Paemeleire, M. Linde, M. Braschinsky, D. Mitsikostas, R. Gil-Gouveia, Z. Katsarava, on behalf of the European Headache Federation and Lifting The Burden: the Global Campaign against Headache

**Affiliations:** 1 Department of Neuromedicine and Movement Science, Norwegian University of Science and Technology, Edvard Griegs Gate, Trondheim, Norway; 2 Division of Brain Sciences, Imperial College London, London, UK; 3 Kiel Headache Centre, Kiel Neurological Pain and Headache Centre, Kiel, Germany; 4 Danish Headache Centre, Department of Neurology, University of Copenhagen, Rigshospitalet-Glostrup Hospital, Glostrup, Denmark; 5 Headache Medical Centre, Ordensklinikum Linz Barmherzige Schwestern, Linz, Austria; 6 Department of Neurology, Ghent University Hospital, Ghent, Belgium; 7 Headache Clinic, Neurology Clinic, Tartu University Clinics, Tartu, Estonia; 8 Neurology Department A, Aeginition Hospital, National & Kapodistrian University of Athens, Athens, Greece; 9 Hospital da Luz Headache Center, Lisbon, Portugal; 10 Evangelical Hospital Unna, Unna, Germany; 11 Department of Neurology, University of Duisburg-Essen, Essen, Germany; 12 EVEX Medical Corporation, Tbilisi, Georgia; 13 IM Sechenov First Moscow State Medical University (Sechenov University), Moscow, Russian Federation

**Keywords:** Headache, Health care, Health service organization, Structured headache services, Specialized headache centres, Service quality, Standards, European Headache Federation, Global Campaign against Headache

## Abstract

In joint initiatives, the European Headache Federation and *Lifting The Burden* have described a model of structured headache services (with their basis in primary care), defined service quality in this context, and developed practical methods for its evaluation.

Here, in a continuation of the service quality evaluation programme, we set out ten suggested role- and performance-defining standards for specialized headache centres operating as an integral component of these services. Verifiable criteria for evaluation accompany each standard. The purposes are five-fold: (i) to inspire and promote, or stimulate the establishment of, specialized headache centres as centres of excellence; (ii) to define the role of such centres within optimally structured and organized national headache services; (iii) to set out criteria by which such centres may be recognized as exemplary in their fulfilment of this role; (iv) to provide the basis for, and to initiate and motivate, collaboration and networking between such centres both nationally and internationally; (v) ultimately to improve the delivery and quality of health care for headache.

## Introduction

Headache services must provide health care to very large numbers of people [1], whose illnesses are the second-highest cause of disability worldwide [2, 3]. At the same time, unless they aspire to high quality in this purpose, such services are likely to be not only inefficient and wasteful of resources but also ineffectual.

In a joint initiative, the European Headache Federation (EHF) and *Lifting The Burden* (LTB) have clarified what this means, defining “headache service quality” [4, 5] and developing methods for its measurement (service quality evaluation: SQE) [6]. These organizations have also described a headache services model – structured, with their base in primary care but with an important and specific role allotted to specialized headache centres [1].

In many countries, however, this role is poorly distinguished: headache centres exist but, performing outside the role boundaries, fail to fulfil it. Here we set out suggested role- and performance-defining standards for specialized headache centres. In doing so, we stress that it is *not* part of our purpose to set criteria for *quality of clinical care*. These are properly determined at a national level, and our proposals assume that they are met. Our focus is on recommendations for service organization, service quality, professional education and research endeavour that specialized headache centres might follow. Beyond these, we suggest criteria by which specialized headache centres might be generally recognized, nationally and/or internationally, as centres of excellence in the headache community.

We also make clear that neither EHF nor LTB offers itself as a certifying agency, providing accreditation for centres judged to fulfil these criteria. National authorities and other competent agencies might nonetheless, and we hope they will, view the standards set out here as a sound basis for accreditation.

Accordingly, the initiative has five purposes:

- to inspire and promote, or stimulate the establishment of, specialized headache centres as centres of excellence;
- to define the role of such centres within optimally structured and organized national headache services;
- to set out criteria by which such centres may be recognized as exemplary in their fulfilment of this role;
- to provide the basis for, and to initiate and motivate, collaboration and networking between such centres both nationally and internationally;
- ultimately to improve the delivery and quality of health care for headache.

## Background: the need for structured headache services

Headache disorders, especially migraine and tension-type headache (TTH), are common and collectively cause substantial levels of public ill health and disability [2, 7, 8]. Yet, throughout the world, they are under-recognized [9]. This misperception is not easily explained, but it is now slowly changing. On the initiative of LTB and the Global Campaign against Headache [10–13], new studies are filling the gaps in our knowledge of the burdens attributable to headache disorders [14–25], which had embraced half the world [7]. These studies confirm, in all regions of the world, that these burdens weigh heavily not only on people with headache but also on their families, friends, work colleagues and, ultimately, society itself. The *Atlas of Headache Disorders* published by the World Health Organization (WHO) in collaboration with LTB, although not a population-based survey, collates corroborative evidence on the impact of headache from over 100 countries [9]. The Global Burden of Disease study has found not only that TTH and migraine are respectively the second and third most prevalent disorders in the world [26] but also that migraine is the second most disabling [2, 3].

The recognizable consequences not only of public ill health but also of high socioeconomic cost [8], coupled with the large numbers of people affected by headache disorders, give rise to the need for organized, structured and adequately-resourced health services to alleviate them [1, 9, 27]. Effective and cost-effective treatments exist for most people with headache [28]; however, they often fail to reach those who need them [9, 29]. Delivering these treatments is, from any sensible perspective, a public-health priority [3, 9]. The indirect costs of headache, arising mostly from lost productivity secondary to disability, vastly outweigh direct treatment costs [8]; consequently, from a societal perspective, headache-untreated costs a great deal more than headache-treated [9]. Even if importance is not attached to the individual burdens attributable to headache [30], society should wish to mitigate the huge financial burden upon itself which headache imposes [9].

Yet, fully developed headache services consume significant health-care resources, and this calls loudly for built-in efficiency with close attention to cost-effectiveness [4–6]. How headache services should be organized with these essentials in mind has been addressed by EHF and LTB in an earlier collaboration [1, 27].

## Organization of headache services

While headache disorders are prevalent and ubiquitous, they manifest extremely variably: at one end of the spectrum is mild episodic TTH occurring a few times a year; at the other are highly disabling disorders such as cluster headache and chronic migraine. Not everyone with headache will benefit from, or therefore needs, the same level of care: for this reason, a stratified system is necessary in which, for equity as well as efficiency, specialized care is reserved for and thereby kept available to those who need it.

The three-tier service-organization model of EHF and LTB [1] is summarized below. National modifications may be demanded to align with existing health-care systems and according to resources, but the model has considerable flexibility that allows adaptation without altering its intrinsic structure.

### Level 1. General primary care

Primary care should be the accessible front line for almost all people with headache disorders. At this level, non-specialists – with some training in headache – should meet the needs of the great majority of people consulting for headache [1], controlling flow to higher levels.

At level 1, most cases of migraine and TTH should be competently diagnosed and managed [1]. Cluster headache, medication-overuse headache (MOH) and some other common secondary headache disorders should be recognized but not necessarily managed; red-flag warnings of serious secondary headaches should also be recognized and duly acted upon. Referral channels to levels 2 and 3, urgent when necessary, should be in place for these cases, and for patients who are diagnostically complex or difficult to manage [1].

This level should also continue long-term care of patients discharged with treatment plans from levels 2 or 3 [1].

### Level 2. Special-interest headache care

Level 2 may, in some countries, be in primary care, provided by general practitioners with a special interest and additional training. In others it is more likely to be offered in polyclinics or district hospitals by neurologists, also with training in headache [1]. Physicians at this level should provide more skilled ambulatory care to most patients referred upwards from level 1 [1].

Their competence should embrace the diagnosis and management of more difficult cases of primary headache and some secondary headache disorders, but not those that are very rare [1]. To fulfil their role, they need access to other services such as neuroimaging, psychology and physiotherapy. For a minority of their patients (perhaps 1% of all headache patients [1]), they require a referral channel to level 3 (Table 1).

**Table 1:** Patients likely to be referred to level 3 (adapted from [1])

Patients with: • refractory disabling headache of any type; • cluster headache and other trigeminal autonomic cephalalgias, at first presentation; • MOH involving drugs of dependence, where personality mitigates against successful withdrawal of medication, or where withdrawal attempts have failed; • high and low CSF-pressure headaches; • trigeminal and other cranial neuralgias or painful lesions of the cranial nerves; • rare primary or secondary headaches; • headaches with severe physical and/or psychological comorbidities. Cases: • of persisting diagnostic uncertainty; • where risk of serious underlying disorders demands specialist investigation; • of other probable or certain serious secondary headache. Patients who may participate in specific level-3 research projects (including clinical trials).	

### Level 3. Specialized headache centres

Specialized headache centres are recommended as tertiary referral centres, providing specialist care to patients with primary or secondary headache disorders that are difficult to diagnose or treat, refractory or rare, or for other reasons require specialist intervention (Table 1) [1, 31–36]. Patients at level 3 should be a very small subset of patients first seen at level 1 and referred upwards, either via level 2 or directly (and urgently when necessary) [1]; additionally, a few may come from the emergency room.

Centres at this level should be nationally-recognized centres of excellence for care, education and research within the headache field; they should concentrate experience in rare primary or secondary headache disorders and cranial neuralgias, and be innovators and/or early adopters of new technologies. They should employ headache specialists and/or neurologists (in either case accredited, when a national accreditation system exists), and be within or closely affiliated to a university or other major hospital with formal academic links [1]. They should offer 24-h inpatient facilities, and have multidisciplinary management competencies. Access to specialists in all other medical fields should provide for the diagnosis and management of the underlying causes of all secondary headache disorders [1].

## The role of specialized headache centres

It is self-evident that specialized headache centres have a role within structured headache services (see Table 2). Nevertheless, in a world with limited resources, and one in which headache wrongly but stubbornly has low priority among calls on these resources [9], this role needs both definition and quantification. Too few specialized centres would not meet need; too many would consume resources that would be better (*ie*, more cost-effectively) spent at lower levels.

**Table 2:** The role of specialized headache centres within structured headache services

• to provide best possible level-3 clinical care for adults and/or children, having regard to the resources locally available; • to support levels 1 and 2 through medical advice; • to provide training in headache to health-care practitioners at all levels; • to contribute to the development and/or periodic review and updating of national management guidelines; • to conduct research into headache of international value and/or appropriate to the needs of the local community; • to provide empirical evidence in support and justification of their existence.	

Provided that levels 1 and 2 are adequately set up also, demand at level 3 (Table 1) should be limited to a very small minority of all people needing health care for headache [1, 27]. It should also be self-regulating, since levels 1 and 2, if adequately set up, will have shorter waiting lists. In reality, headache services are not well structured in most countries [9, 27], and evolution towards better organization is slow. Change requires an evidence base, and few centres have justified their existence by documenting their activities and outcomes in pursuit of their role [31–35, 37–39]. Efficient achievement of desired outcomes is what justifies investment, so demonstration of this is a requirement not just for the continued existence of established specialized centres but also for appropriate expansion in their number.

This is the context in which the need arises for standards for specialized headache centres: standards that are universally applicable despite variations in health-care systems among nations, and maintained by international collaborative networks between centres – themselves made possible by adoption of these standards.

## Standards and criteria

The purpose of standards is to encourage excellence. Criteria are the yardstick by which excellence may be recognized. Criteria, therefore, must be verifiable.

They must also be pragmatic rather than absolute. In less-well-resourced countries, ideals may not be achievable, yet performance backed by aspiration may nonetheless be meritorious in the context, and highly worthy of encouragement.

Table 3 sets out a suggested template for centre evaluation against these standards. It sets out aspirational targets: no benchmarks are proposed, because it is not yet clear what these should be.

**Table 3:** Suggested assessment template

Standard		Criterion	Verification	Target
Domain A. Competence of staff				
1	Centre is staffed by headache specialists, who are sufficient but not excessive in number.	Each “headache specialist” can document:(a) advanced training (yes/no); (b) past and continuing experience in the field of headache (yes/no). Number of specialists is: (c) sufficient (yes/no); (d) not excessive (yes/no).	(a, b) internal audit of CVs and continuing professional development records; (c, d) internal audit of workload and waiting times	(a) 100% yes (b) 100% yes (c) aspirational (d) yes
Domain B. Provision of care				
2	Centre provides dedicated care for headache patients.	Patients with headache are seen in dedicated sessions, not within general neurological or other sessions (yes/no)	internal audit of clinic lists	yes as a general rule
3	Centre provides patients with a clear diagnosis made at earliest opportunity, information about their headache(s), advice on management and internationally-accepted evidence-based treatment.	(a) Diagnoses are always according to ICHD-3 (yes/no). Disability assessments, diagnostic and follow-up diaries, outcome measures and patient information leaflets are: (b) all available (yes/no): (c) all routinely used (yes/no). (d) National or international management guidelines are adopted (yes/no).	(a) internal audit of patients’ records; (b) objectively verifiable; (c) internal audit of patients’ records; (d) objectively verifiable	(a) 100% yes (b) yes (c) yes (d) yes
4	Centre provides multidisciplinary care full-time, and competently manages disorders underlying the full range of secondary headaches.	(a) Working collaborations exist between physicians, nurses, physical therapists and psychologists (yes/no). (b) The centre is based within, or in geographical proximity to, a general hospital providing access to emergency department, neurology, neuroradiology, neurosurgery, psychiatry, ophthalmology, otorhinolaryngology, orthopaedics, rheumatology, cardiology, infectious diseases, endocrinology, paediatrics, gynaecology, dentistry (yes/no). (c) Inpatient facilities are available for patients with certain comorbidities and for those needing supervised withdrawal from medication overuse (yes/no).	(a) existence is objectively verifiable; (b) access is objectively verifiable; (c) availability is objectively verifiable	(a) yes (b) yes (c) yes
Domain C. Quality evaluation and assurance				
5	Centre monitors quality of care in order to optimize it.	Procedures are in place for recording clinical outcomes and adverse events, and service quality indicators (Table 4), with regular audits of all (yes/no).	objectively verifiable as present and happening	yes
Domain D. Networks and collaborations				
6	Centre maintains quality of endeavour through networking, collaboration and the sharing of experience with other international and/or national centres.	(a) Existence and operation of networks and collaboration are documented by the centre (yes/no). (b) Evidence is presented of any of the following (yes/no): • exchange of ideas relating to service organization, patient care, teaching and/or research; • exchange of staff and/or engagement in a fellowship exchange programme; • collaborative research protocols; • shared or collaborative educational programmes; • shared or common database.	(a, b) verifiable by peer review	(a) yes (b) yes to one or more
Domain E. Teaching				
7	Centre is a principal resource for national postgraduate training in the field of headache.	Evidence is presented of recent or current engagement in at least two of the following (yes/no): • development of national management guidelines, or adaptation of international guidelines for national use; • development of learning materials for trainee headache specialists, neurologists and/or specialist nurses; • delivery of didactic teaching and/or clinical demonstrations to trainee headache specialists, neurologists and/or specialist nurses on a regular basis; • acceptance of clinical trainees on accredited attachments.	verifiable by peer review	yes to two or more
8	Centre provides support, through training and education, to health-care providers at levels 1 and 2.	Either: (a) a programme of training and education is offered through formal links between the centre and health-care providers at levels 1 and 2 throughout the geographical area served by the centre (yes/no); (b) where levels 1 and 2 are not in place within structured services, a programme of training and education is continuously available to local general practitioners, nurses and/or pharmacists (yes/no)	(a) objectively verifiable as in place; (b) verifiable by peer review	yes to either
Domain F. Research				
9	Centre is a principal fount of useful research output in the field of headache.	Research is either or both: (a) of international value (yes/no); (b) appropriate to the needs of the local community (yes/no).	peer review of quantity, quality and value of publications	yes to either
Domain G. Empirical support of existence				
10	Centre supports and justifies its existence, and the development of others, by documenting and demonstrating its utility.	Activities and achievements: (a) are documented (yes/no); (b) provide evidence of utility (yes/no).	(a) objectively verifiable; (b) verifiable by peer review	(a) yes (b) yes

## Competence of staff

### Standard 1

A centre of excellence is staffed by headache specialists, who are sufficient but not excessive in number.

#### Criteria

1. A “headache specialist” should be able to document (a) advanced training and (b) past and continuing experience in the field of headache. These are objectively verifiable.
  National certification in neurology is not sufficient. Requisite expertise may be self-evident in those with long experience in headache care, whose status and credentials are widely acknowledged and who may be national or international leaders in the field. Otherwise, it may be acquired through recognized training: for example, through full-time attachment for one year to a level-3 centre, or specific training programmes such as the Master Degree courses at Sapienza University of Rome [40–42] or the Danish Headache Centre, University of Copenhagen [43].
2. Sufficiency in number is an obvious requirement for effective operation, whereas excess (in practice unlikely) is wasteful of resources.
  What constitutes sufficiency without excess must be determined locally in accordance with how services are organized [1]. This part of the standard is aspirational. In a world of limited resources, a centre may still be recognized as exemplary despite that it is struggling against an excessive workload.

## Provision of care

### Standard 2

A centre of excellence provides dedicated care for headache patients.

#### Criterion

Patients with headache are, as a rule, seen in dedicated sessions, not within general neurological or other sessions. This is objectively verifiable.

### Standard 3

A centre of excellence provides patients with a clear diagnosis made at the earliest opportunity, information about their headache(s), advice on management and internationally-accepted evidence-based treatment.

#### Criteria

1. Diagnoses are always according to the *International Classification of Headache Disorders* (ICHD) (ICHD-3 [44], the latest edition, aligns with WHO’s International Classification of Diseases, 11th revision [ICD-11] [45]). This criterion is objectively verifiable.
2. The centre has available and routinely makes use of disability assessments, diagnostic and follow-up diaries, outcome measures and patient information leaflets (such as those published by LTB [28, 46]). This is objectively verifiable.
3. The centre has adopted national or international management guidelines. This is objectively verifiable.

Acceptable national management guidelines are those adopted by national professional organizations or by health authorities. Where none exist, any published international guidelines may be followed (adapted, to the extent necessary, according to availability of treatments).

### Standard 4

A centre of excellence provides full-time multidisciplinary care and (or with access to) round-the-clock inpatient facilities, and competently manages disorders underlying the full range of secondary headaches.

“Full-time” means on a daily basis, not necessarily 24 h per day, seven days per week.

Capacity in all of these should be sufficient, in the local conditions, to obviate undue treatment delays, but this again is aspirational.

#### Criteria

1. Multidisciplinary care is provided through working collaborations between physicians, nurses, physical therapists and psychologists. Existence of these collaborations is objectively verifiable.
  Physical therapists and psychologists collaborating with the service need not be employed within it.
2. There is access to a full range of other specialists. This is objectively verifiable.
  Accessible specialties should include at least the following: neurology, neuroradiology, neurosurgery, psychiatry, ophthalmology, otorhinolaryngology, orthopaedics, rheumatology, cardiology, infectious diseases, endocrinology, paediatrics, gynaecology and dentistry. This requirement implies that the centre is within or works very closely with and in geographical proximity to a general hospital offering an emergency department.
3. Inpatient facilities, on-site or nearby, are accessible for the care and management of patients with certain comorbidities, and for those needing supervised withdrawal from medication overuse. This is objectively verifiable.

## Quality evaluation and assurance

### Standard 5

A centre of excellence monitors quality of care in order to optimize it.

#### Criterion

Procedures are in place for recording clinical outcomes and adverse events, and service quality indicators according to Table 4 [5]. All are audited regularly as part of quality assurance. These are objectively verifiable as present and happening, or not.

**Table 4:** Domains and indicators of quality in headache service delivery (from [5])

Domain A. Accurate diagnosis is essential for optimal headache care	
A1	Patients are asked about onset of their headaches
A2	Diagnosis is according to current ICHD criteria
A3	A working diagnosis is made at the first visit
A4	A definitive diagnosis is made at first or subsequent visit
A5	Diagnosis is reviewed during later follow-up
A6	Diaries are used to support or confirm diagnosis
Domain B. Individualized management is essential for optimal headache care	
B1	Waiting-list times for appointments are related to urgency of need
B2	Sufficient time is allocated to each visit for the purpose of good management
B3	Patients are asked about the temporal profile of their headaches
B4	Treatment plans follow evidence-based guidelines, reflecting diagnosis
B5	Treatment plans include psychological approaches to therapy when appropriate
B6	Treatment plans reflect disability assessment
B7	Patients are followed up to ascertain optimal outcome
Domain C. Appropriate referral pathways are essential for optimal headache care	
C1	Referral pathway is available from primary to specialist care
C2	Urgent referral pathway is available when necessary
Domain D. Education of patients about their headaches and their management is essential for optimal headache care	
D1	Patients are given the information they need to understand their headache and its management
D2	Patients are given appropriate reassurance
Domain E. Convenience and comfort are part of optimal headache care	
E1	The service environment is clean and comfortable
E2	The service is welcoming
E3	Waiting times in the clinic are acceptable to both health-care providers and patients
Domain F. Achieving patient satisfaction is part of optimal headache care	
F1	Patients are satisfied with their management
Domain G. Optimal headache care is efficient and equitable	
G1	Procedures are followed to ensure resources are not wasted
G2	Patients are not over-investigated
G3	Costs of the service are measured as part of a cost-effectiveness policy
G4	There is equal access to headache services for all who need it
Domain H. Outcome assessment is essential in optimal headache care	
H1	Outcome measures are based on self-reported symptom burden (headache frequency, duration and intensity)
H2	Outcome measures are based on self-reported disability burden
H3	Outcome measures are based on self-reported quality of life
Domain I. Optimal headache care is safe	
I1	Patients are not over-treated
I2	Systems are in place to be aware of serious adverse events

## Networks and collaborations

### Standard 6

Centres of excellence maintain quality of endeavour through networking, collaboration and the sharing of experience with other internationally- and/or nationally-recognized centres.

The nationwide German headache treatment network provides good examples of networking and collaboration at national [33, 34] and international [36] levels.

#### Criteria

1. The existence and operation of networks and collaboration are documented by the centre. This is verifiable by peer review.
2. Evidence is presented of any or all of the following:
  exchange of ideas relating to service organization, patient care, teaching and/or research;
  exchange of staff and/or engagement in a fellowship exchange programme;
  collaborative research protocols;
  shared or collaborative educational programmes;
  shared or common database.

These are verifiable by peer review.

## Teaching

### Standard 7

Centres of excellence are a principal resource for national postgraduate training in the field of headache.

#### Criterion

Evidence is presented of recent or current engagement in at least two of the following:

- development of national management guidelines, or adaptation of international guidelines for national use;
- development of learning materials for trainee headache specialists, neurologists and/or specialist nurses;
- delivery of didactic teaching and/or clinical demonstrations to trainee headache specialists, neurologists and/or specialist nurses on a regular basis;
- acceptance of clinical trainees on accredited attachments.

These are verifiable by peer review.

### Standard 8

Centres of excellence delivering level-3 care within structured headache services also provide support, through training and education, to health-care providers at levels 1 and 2.

It is understood that, in some countries or areas, levels 1 and 2 may not be in place.

#### Criterion

Either of (a) or (b):

- Formal links exist between the centre and health-care providers at levels 1 and 2 throughout the geographical area served by the centre. Through these links, a programme of training and education is offered. The existence of these is objectively verifiable.
- Where levels 1 and 2 are not in place within structured services, a programme of training and education is continuously available to local general practitioners, nurses and/or pharmacists. The existence of this alternative is verifiable by peer review.

## Research

### Standard 9

Centres of excellence are a principal fount of useful research output in the field of headache.

#### Criterion

Research by the centre is of international value and/or appropriate to the needs of the local community. The quantity, quality and value of publications are verifiable by peer review.

Research may include development and maintenance of patient databases, public-health initiatives, epidemiology, pathophysiological and other clinical research, evaluation of diagnostic and therapeutic guidelines and therapeutic trials into which there is intellectual input. Simple recruitment into industry-sponsored trials does not fulfil this criterion.

## Empirical support of existence

### Standard 10

Centres of excellence support and justify their existence, and the development of others, by documenting their activities and achievements and demonstrating their utility within structured headache services.

#### Criteria

1. Activities and achievements are documented. This is objectively verifiable.
2. Activities and achievements provide evidence of utility. This is verifiable by peer review.

## Conclusions

Ten standards are defined, each one accompanied by one or more verifiable criteria. Collectively these define the role of specialized headache centres within structured headache services, and allow for evaluation of performance as part of service-quality assurance. Their adoption should, ultimately, improve the delivery and quality of health care for headache.

Agencies with appropriate competence and authority might use these standards as a basis for centre-accreditation.

## Abbreviations

CSF: Cerebrospinal fluid
EHF: European Headache Federation
ICD: International Classification of Diseases
ICHD: International Classification of Headache Disorders
LTB: *Lifting The Burden*
MOH: Medication-overuse headache
SQE: Service-quality evaluation
TTH: Tension-type headache
WHO: World Health Organization
